# Survival of Patients with Oral Cavity Cancer in Germany

**DOI:** 10.1371/journal.pone.0053415

**Published:** 2013-01-18

**Authors:** Stefan Listl, Lina Jansen, Albrecht Stenzinger, Kolja Freier, Katharina Emrich, Bernd Holleczek, Alexander Katalinic, Adam Gondos, Hermann Brenner, the GEKID Cancer Survival Working Group

**Affiliations:** 1 Department of Conservative Dentistry, University Hospital Heidelberg, Heidelberg, Germany; 2 Munich Center for the Economics of Aging, Max-Planck-Institute for Social Law and Social Policy, Munich, Germany; 3 Division of Clinical Epidemiology and Aging Research, German Cancer Research Center, Heidelberg, Germany; 4 Institute of Pathology, University Hospital Heidelberg, Heidelberg, Germany; 5 Department of Oral and Maxillofacial Surgery, University Hospital Heidelberg, Heidelberg, Germany; 6 Cancer Registry of Rhineland-Palatine, Institute for Medical Biostatistics, Epidemiology and Informatics, University Medical Center, Johannes Gutenberg University Mainz, Mainz, Germany; 7 Saarland Cancer Registry, Saarbrücken, Germany; 8 Institute of Clinical Epidemiology, Lübeck, Germany; Baylor College of Medicine, United States of America

## Abstract

The purpose of the present study was to describe the survival of patients diagnosed with oral cavity cancer in Germany. The analyses relied on data from eleven population-based cancer registries in Germany covering a population of 33 million inhabitants. Patients with a diagnosis of oral cavity cancer (ICD-10: C00-06) between 1997 and 2006 are included. Period analysis for 2002–2006 was applied to estimate five-year age-standardized relative survival, taking into account patients' sex as well as grade and tumor stage. Overall five-year relative survival for oral cavity cancer patients was 54.6%. According to tumor localization, five-year survival was 86.5% for lip cancer, 48.1% for tongue cancer and 51.7% for other regions of the oral cavity. Differences in survival were identified with respect to age, sex, tumor grade and stage. The present study is the first to provide a comprehensive overview on survival of oral cavity cancer patients in Germany.

## Introduction

Oral cavity cancer is one of the more frequent cancer types worldwide [1], [2]. According to ICD-10, oral cavity cancer includes cancer of the lip (ICD-10: C00), cancer of the tongue (ICD-10: C01-02), and cancer within other regions of the oral cavity (ICD-10: C03-06). Most oral malignancies are squamous cell carcinomas and the risk of developing oral cancer has been shown to generally increase with age [3]. Amongst Europeans, the lifetime risk of developing oral and pharyngeal cancer was recently estimated to be 1.85% for men and 0.37% for women [4]. Tobacco use and excess consumption of alcohol have been suggested to be important etiological factors whose elimination could decisively reduce the number of oral cancer cases [5].

Despite recent clinical improvements of diagnosis and treatment [6]–[8], the prognosis is still mostly dependent on the point of time at which oral cavity cancer is detected. Above all, no major improvement in survival has been reported from population-based survival studies in the past decades [3], [9]. Dental professionals' responsibility in monitoring oral cavity cancer is, thus, paramount. Moreover, information about population level survival and their determining parameters is pivotal for public health decision makers who seek to understand how prevention and treatment of oral cavity cancer can further be improved. So far, however, there is only little epidemiological evidence about the survival of patients diagnosed with oral cavity cancer in Germany. Until recently, population-based cancer survival analysis in Germany had to rely mostly on data from Saarland which includes about 1 million inhabitants, thus covering only about 1.3% of the total German population [10], [11]. In international comparative studies (EUROCARE; EUNICE), Germany was only represented by Saarland and, sometimes, by the Munich Cancer registry [12], [13].

The aim of the present study was, therefore, to provide up-to-date and detailed cancer survival estimates for patients who were diagnosed with cancer of the lip (ICD-10: C00), tongue (ICD-10: C01-02) and other parts of the oral cavity (ICD-10: C03-06) based on a much larger database covering about 33 million inhabitants in Germany.

## Methods

### Data sources

The present analysis includes patients diagnosed between 1997 and 2006 with cancer of the lip (ICD-10: C00), tongue (base of tongue: ICD-10: C01; other/unspecified part of tongue: ICD-10: C02), and other parts of the oral cavity (gum: ICD-10: C03; floor of mouth: ICD-10: C04; palate: ICD-10: C05; other/unspecified part of the mouth: ICD-10: C06). The data set includes data from eleven cancer registries in Germany, covering a population of 33 million people, which fulfill the following criteria: percentage of death certificate only (DCO) cases for all cancer sites combined below 20% throughout the study period or decrease by at least two percentage points per year to levels below 20% by the end of the study period. The latter criterion was introduced since high percentages of DCO cases can occur in “younger” cancer registries who may have achieved a high level of case ascertainment within a relatively short time period. For the states of Bavaria, Schleswig-Holstein and Rhineland-Palatinate, data were used from those administrative regions where these criteria were met. For the cancers assessed in this analysis, DCO proportions were below 13% in all included registries (7.3% overall). Estimated completeness of cancer registration was over 80% in all included states and over 90% in most included states in 2004–2006 [14]. Specific details of the data sources and quality control checks are described elsewhere [15]. Follow-up of vital status was performed until the end of 2006. Cancer topography, morphology, and behavior were originally coded in accordance with the ICD-O-3 (International Classification of Diseases for Oncology) and later converted into ICD-10 using “IARC crgTools” [16]. Cases identified on basis of DCO and autopsy only were excluded from the analysis.

### Statistical Methods

Five-year relative survival estimates were calculated for the time period 2002–2006 which corresponds to the most recent 5-year interval for which data were available. The according values represent cancer-attributable excess mortality in the underlying population and are computed as the ratio between the survival in the patient group and the expected survival of a comparison group in the general population [17]. Expected relative survival was estimated according to the Ederer II method [18] by means of life tables stratified by age, sex, and calendar period as provided by the German Federal Statistical Office. The applied method of period analysis is particularly appealing for cancer survival analysis because it facilitates more up-to-date estimates of cancer survival than traditional cohort-based studies [19], [20].

For cancers of the oral cavity (ICD-10: C00-06), five-year age-standardized relative survival estimates were calculated in accordance with the International Cancer Survival Standards (ICSS). ICSS age groups are defined as: 15–44 years; 45–54 yrs; 55–64 yrs; 65–74 yrs; and 75+ years [21]. Relative survival estimates by age, sex, stage and grade were presented for overall oral cavity cancer, as well as cancer of the lip, tongue, and other parts of the oral cavity. In addition, model-based period analysis [22] was used to test for sex differences in five-year relative survival once with adjustment for age and once with adjustment for age and stage. In this model-based period analysis, numbers of deaths were modelled as a function of year of follow-up (categorical variable), age (ICSS age groups, categorical), sex, and, depending on the model, clinical stage (local, regional, distant) with the logarithm of the person-years at risk as an offset. All data analysis in the present study was carried out with the software package SAS 9.2 (SAS Institute, Cary, NC, USA), using macros developed for period analysis [23] and model-based period analysis [22]. Statistical significance was defined by p<0.05. No multiple comparison corrections were made.

## Results

Table 1 shows the numbers of cases submitted by each cancer registry, together with the underlying population in 2006, i.e. about 33 million persons overall. After exclusion of cancers notified by death certificates only and based on autopsy, the dataset included 15,792 cases of oral cavity cancer (ICD10: C00-06) diagnosed in Germany between 1997 and 2006. Of these cases, 7.5% were localized in the lip (ICD-10: C00), 39.5% in the tongue (ICD-10: C01-C02), and 53.0% in other parts of the oral cavity (ICD-10: C03-C06). The median age at diagnosis ranged between 59 and 62 years. Across all cancer registries, the percentage of DCO cases was 7.3% and the percentage of histologically confirmed oral cavity cancer cases amounted to 99.0%.

**Table 1:** Description of the dataset used for period survival analysis of patients diagnosed with oral cavity cancer in Germany, 1997–2006.

Region	Underlying population in 2006 (Million)	Diagnosis period included	Cases	Exclusion based on DCO or autopsy (%)	Cases included in the analysis	Median age at diagnosis	Histologically confirmed cases (%)
**Bavaria**	8.13	2002–2006	2688	6.8	2504	59	100
**Brandenburg**	2.55	1997–2006	1655	6.7	1543	60	99.1
**Bremen**	0.66	1998–2006	571	6.7	533	62	99.1
**Hamburg**	1.75	1997–2006	1321	10.3	1185	61	96.9
**Mecklenburg-Vorpommern**	1.69	1997–2006	1359	5.7	1281	59	99.8
**Lower Saxony**	7.98	2001–2006	2832	12.4	2480	62	97.9
**North Rhine-Westphalia**	2.62	1998–2004	1154	6.8	1076	61	97.9
**Rhineland-Palatinate**	0.52	1998–2006	364	9.9	328	61	100
**Saarland**	1.04	1997–2006	1027	0.5	1022	60	99.8
**Saxony**	4.25	1997–2006	2776	3.4	2682	59	99.4
**Schleswig-Holstein**	1.85	1999–2006	1293	10.4	1158	61	99.9
**Total**	33.04		17040	7.3	15792	60	99.0

**NB:** for Bavaria, Schleswig-Holstein, Rhineland-Palatinate, and North Rhine-Westphalia, only selected administrative regions met the inclusion criteria.

Table 2 reports relative survival estimates for all oral cavity cancer cases (ICD-10: C00-C06). The age-standardized overall five-year relative survival amounted to 54.6%. Cancer survival was associated with patient's age. Grouping both sexes together yielded a pattern in which survival decreased from 60.6% to 51.0% between patients aged 15–44 and 65–74 years but increased thereafter to a level of 59.3%. If separated according to sex, the described discontinuity between decreasing survival until age 74 and increase thereafter only remained for men. In contrast, a steady decrease in cancer survival with age was observed for women. In general, five-year relative survival was higher for women than for men overall (women: 61.3%, men: 53.0%, respectively) and in all age groups, except for persons aged 75+. Both tumor stage and grade were associated with survival: it decreased from 70.9% for patients with local stage tumors of the oral cavity to 6.4% for those with metastases at first diagnosis and from 75.6% for grade 1 tumors to 43.9% for grade 3 and 4 tumors. In a further step (not shown in table 2), model-based period analysis revealed by 10.9% points higher survival for women than men if adjusting for age only (p<0.001) and by 8.5% points higher survival for women than men if adjusting for age and stage (p<0.001).

**Table 2:** Overall five-year relative survival of patients with oral cavity cancer (ICD-10: C00-C06) in Germany (period analysis, 2002–2006).

			Overall			Male			Female	
Factor	Level	N	5-year RS	SE	N	5-year RS	SE	N	5-year RS	SE
**Age**	15–44	1307	60.6	1.9	1025	58.3	2.2	282	68.7	3.8
	45–54	3754	52.9	1.2	3017	49.2	1.3	737	67.5	2.4
	55–64	5054	52.0	1.1	3938	48.7	1.2	1116	63.3	2.1
	65–74	3697	51.0	1.3	2674	47.7	1.6	1023	59.3	2.4
	75+	1980	59.3	2.5	983	61.8	3.6	997	57.2	3.4
**Stage** a	Local	3871	70.9	1.9	2797	74.0	2.8	1074	72.2	2.8
	Regional	4714	37.2	1.5	3654	34.3	1.9	1060	43.7	2.5
	Distant	373	6.4	1.7	292	6.3	1.9	81	6.3	3.7
	Unknown^b^	6832	57.7	1.2	4892	55.0	1.7	1940	66.1	1.9
**Grade** a	1	1677	75.6	2.3	1095	79.4	3.4	582	76.1	3.3
	2	8896	54.3	1.2	6663	52.5	1.6	2233	61.3	1.9
	3 and 4	3159	43.9	2.1	2434	41.0	2.7	726	50.5	3.5
	Unknown	2060	51.1	2.2	1445	48.3	3.0	614	59.5	3.3
**All** a		15792	54.6	0.9	11637	53.0	1.2	4155	61.3	1.4

**NB:** RS = Relative survival SE = Standard error;

a Age-standardized estimates;

b 2 male patients with wrong stage code were excluded.

Table 3 shows relative survival estimates for patients with cancers of the oral cavity by subsite. The overall estimate for five-year relative survival regarding cancer of the lip (ICD-10: C00) was 86.5%. Survival decreased from 85.7% in patients aged 15–64 years to 79.9% in patients aged 65–74 years and, finally, increased again to 93.8% for patients aged 75 years or older. Age-standardized relative survival was higher for men (88.1%) than for women (81.2%). Tumor stage and grade contributed to level of survival: it varied between 86.7% (local stage) and 62.0% (regional stage) as well as between 93.0% (grade 1) and 70.9% (grade 3 and 4). In a further step (not shown in table 3), model-based period analysis no significant sex differences were observed after adjusting for age or for age and stage (p = 0.543 and 0.561, respectively).

**Table 3:** Five-year relative survival for patients with cancer of the lip (ICD-10: C00), cancer of the tongue (ICD-10: C01-02), and cancer within other parts of oral cavity (ICD-10: C03-06) in Germany (period analysis, 2002–2006).

			Lip			Tongue			Other parts	
Factor	Level	N	5-year RS	SE	N	5-year RS	SE	N	5-year RS	SE
**Age**	15–44	330	85.7	3.1	550	61.1	3.0	729	59.2	2.6
	45–54				1516	51.0	1.9	2149	52.6	1.5
	55–64				2116	49.1	1.6	2725	51.6	1.5
	65–74	449	79.9	3.5	1400	43.9	2.1	1848	49.0	1.8
	75+	404	93.8	5.6	659	47.3	4.1	917	52.1	3.6
**Sex** a	Male	861	88.1	2.9	4631	44.9	2.0	6145	48.8	1.7
	Female	322	81.2	3.4	1610	56.0	2.2	2223	60.4	1.9
**Stage** a	Local	488	86.7	3.9	1408	67.4	3.3	1975	65.5	3.3
	Regional	51	62.0	12.7	2207	36.4	2.2	2456	36.8	2.1
	Distant	5	0.0	0.0	160	6.2	2.5	208	6.9	2.5
	Unknown	639	87.4	3.0	2466	49.3	2.2	3727	55.8	1.8
**Grade** a	1	381	93.0	3.6	495	65.2	5.8	801	70.4	3.6
	2	533	82.0	3.9	3525	48.9	1.9	4838	51.3	1.7
	3 and 4	92	70.9	9.1	1441	41.1	2.9	1547	43.9	3.2
	Unknown	175	88.3	5.7	744	42.6	3.8	1141	48.7	2.9
**All** a		1183	86.5	2.3	6241	48.1	1.4	8368	51.7	1.3

**NB:** RS = Relative survival; SE = Standard error;

a Age-standardized estimates.

The overall estimate for five-year survival of patients with cancers of the tongue (ICD-10: C01-02) was 48.1%. Survival decreased from 61.1% in patients aged 15–44 years to 43.9% in patients aged 65–74 years but increased thereafter to 47.3%. The age-standardized five-year relative survival estimates were much higher for women (56.0%) than for men (44.9%). Tumor stage and grade were strongly related to survival: it varied between 67.4% (local stage) and 6.2% (distant stage) as well as between 65.2% (grade 1) and 41.1% (grade 3 and 4). In a further step (not shown in table 3), model-based period analysis revealed by 11.9% points higher survival for women than men if adjusting for age only (p<0.001) and by 8.6% points higher survival for women than men if adjusting for age and stage (p<0.001).

The overall estimate for five-year relative survival of patients with neoplasms of oral cavity parts other then lip and tongue (ICD-10: C03-06: gum, floor of the mouth, palate, and other unspecified parts of the mouth) was 51.7%. Survival decreased from 59.2% in patients aged 15–44 years to 49.0% in patients aged 65–74 years. No further decrease was seen among older patients. Age-standardized relative survival was higher for women (60.4%) than for men (48.8%). Tumor stage and grade were again strongly related to the level of survival: it varied between 65.5% for local stage tumors and 6.9% for cancers with involvement of distant sites as well as between 70.4% for grade 1 tumors and 43.9% for grade 3 and 4 tumors. In a further step (not shown in table 3), model-based period analysis revealed by 13.4% points higher survival for women than men if adjusting for age only (p<0.001) and by 10.4% points higher survival for women than men if adjusting for age and stage (p<0.001).

The group “other unspecified parts of the mouth” (ICD-10: C03-C06) was further subdivided in cancers of “floor of mouth” (ICD-10: C04) and “gum and other mouth” (ICD-10: C03, C05-C06) in accordance to the grouping used in the SEER data (Table 4) [24]. For cancers of the floor of the mouth (ICD-10: C04; representing 25.9% of oral cancer cases in the sample) five-year age-standardized relative survival was overall 49.3% and generally comparable for women (51.1%) and men (49.1%). Relative survival decreased with age from 55.3% for patients age 15–44 to 45.6% for patients older than 65 years. With respect to stage and grade, survival estimates varied between 63.8% (local stage) and 4.6% (distant stage) as well as between 66.3% (grade 1) and 36.2% (grade 3 and 4). In a further step (not shown in table 4), model-based period analysis survival was 8.9% higher for women than men if adjusting for age only (p = 0.019). If adjusting for stage and age, the survival advantage of women of 4.4 percent units was not significant (p = 0.188). For cancers of the gum or other parts of the mouth (ICD-10: C03, C05, C06), representing 27.1% of oral cancer cases in the sample, five-year age-standardized relative survival of 54.0% was calculated (Table 4). The pattern in survival with respect to age, sex, stage, and grade was comparable with the pattern described for the group “other unspecified parts of the mouth” including additionally cancer in the floor of the mouth (ICD-10 C04).

**Table 4:** Five-year relative survival for patients diagnosed with cancer of the floor of mouth (ICD-10: C04) and cancer of the gum and other mouth (ICD-10: C03, C05, C06) in Germany (period analysis, 2002–2006).

		floor of mouth			gum and other mouth		
Factor	Level	N	5-year RS	SE	N	5-year RS	SE
**Age**	15–44	411	55.3	3.6	318	63.7	3.8
	45–54	1219	50.3	2.0	930	55.9	2.4
	55–64	1438	47.9	2.0	1287	55.6	2.1
	65–74	1027	45.6	2.7	1073	52.8	2.4
	75+				665	50.9	4.2
**Sex** a	Male	3316	49.1	2.7	2829	49.3	2.2
	Female	779	51.1	4.7	1444	63.9	2.2
**Stage** a	Local	1037	63.8	5.3	940	67.0	4.1
	Regional	1278	35.0	4.4	1178	40.1	2.5
	Distant	123	4.6	2.6	85	4.4	2.0
	Unknown^b^	1657	53.8	3.5	2700	58.0	2.1
**Grade** a	1	295	66.3	12.5	506	73.7	3.9
	2	2492	51.7	3.1	2346	51.7	2.1
	3 and 4	826	36.2	5.7	762	49.1	3.9
	Unknown	482	45.1	5.1	659	52.5	3.5
**All** a		4095	49.3	2.4	4273	54.0	1.5

**NB:** RS = Relative survival; SE = Standard error;

a Age-standardized estimates.

## Discussion

This comprehensive population-based study provides detailed and up-to-date data on oral cavity cancer survival in Germany, relying on data of over 15,000 patients diagnosed with oral cavity cancer in 1997–2006 from an underlying population of 33 million people. The according findings indicate an overall five-year age-standardized relative survival of 54.6%. For women, survival generally decreased with age. For men, survival showed a similar pattern up to the age group 65–74 years, but somewhat higher survival among patients of age 75+.

Distinction between different oral localizations proofs relevant: patients with cancer of the lip were shown to have a five-year relative survival of 86.5%. This was almost 40% units higher than the survival of patients with tongue cancer (48.1%), and by about 35% units higher than that of a tumor within other regions of the oral cavity (51.7%). The lowest overall survival was found for cancer in the floor of mouth (49.3%). Only very few lip cancer cases had a distant disease stage or a poorly diffentiated carcinoma (grade 3 or 4) whereas for the tongue as well as other regions of the oral cavity, there was a comparably wide survival range according to tumor stage and grade: Five-year relative survival ranged from more than 60% for localized cancer to less than 10% for cancers with distant metastases. Cancer of the lip also proved as a distinct entity in that it did not exhibit a significant sex difference in age-adjusted cancer survival whereas for the tongue and other regions of the oral cavity age-adjusted cancer survival was significantly higher amongst women than men. The according sex-differences could only partially be explained by differences in tumor stage.

Relative to other developed countries, the results of our study compare as follows: In the US, the overall five-year relative survival (period of diagnosis: 2001–2007; age-standardized estimates according to SEER) for neoplasms of the lip amounted to 90.5%. For cancers of the tongue it was 59.4%. For cancers of the gum and other localizations within the mouth and for cancers of the floor of the mouth five-year relative survival estimates of 58.1% and 51.2% have been reported, respectively [24]. Notably, survival in the US differ significantly with respect to ethnicity (higher survival for “Caucasian” than “Afroamerican” patients). In general, the US data indicate a tendency towards higher survival for patients diagnosed with oral cancer than observed for Germany.

According to NORDCAN, five-year relative survival in Scandinavian countries presents as follows (period of diagnosis: 1999–2003; age-standardized estimates reported separately for men/women): Denmark: neoplasm of lip: 86/88%; neoplasm of tongue: 35/45%; neoplasm of other part of oral cavity: 40/49%; Finland: neoplasm of lip: 94/96%; neoplasm of tongue: 50/68%; neoplasm of other part of oral cavity: 43/64%; Norway: neoplasm of lip: 87/98%; neoplasm of tongue: 48/58%; neoplasm of other part of oral cavity: 54/59%; Sweden: neoplasm of lip: 91/91%; neoplasm of tongue: 46/56%; neoplasm of other part of oral cavity: 51/61% [25].

The overall survival estimates for Germany as reported in the present paper were within a similar range as those from several Scandinavian countries. Our findings of higher survival estimates for patients with cancers of the lip than for cases with cancers of the tongue or other parts of the oral cavity were likewise in agreement with data from both the USA and the Scandinavian countries. For lip cancer, previous literature has not only reported a relatively good surgical operability, but has also shown that most of the cases are localized on the lower lip and are squamous cell carcinomas [26]. Such carcinomas have been reported to have relatively low rates of spread to nearby lymph nodes and distant sites. Lip cancer is therefore perceived as having a relatively good prognosis [27]. Interestingly, there were similar sex differences between Germany and Scandinavia, i.e. differences in survival between men and women were non-significant for lip cancer but significant for the tongue and other localizations of the oral cavity, except for Norway. One potential explanation for the relatively small sex margin among patients with lip cancer may be that, due to its comparably high visibility, a lip cancer is more likely to be identified by persons other than the affected individual. Consequently, this may mitigate eventual duration differences between women and men until medical advice is sought.

While survival for female patients in Germany continuously decreased with increasing age, our study revealed a tentative re-increase of five-year relative survival of men with oral cavity cancer at age 75+ compared to younger patients. It is tempting to speculate whether this survival pattern is attributable to selection effects due to age-related increases of other potentially fatal co-morbidities (e.g. cardiovascular diseases), many of which are closely related to smoking, the key risk factor for oral cavity cancers. In other words, men at age 75+ may respond relatively positively to oral cancer treatment, simply because a high proportion of multi-morbid, smoking men may already have died in earlier life years.

The focus of the present paper has been on cancer of the oral cavity. Earlier reported survival estimates from Germany were mostly restricted to the Saarland registry and to estimates which aggregated oral and pharyngeal cancer [28]–[30]. Previous evidence has shown significant survival differences between oral and pharyngeal cancer [24] which makes a reliable assessment of temporal trends regarding survival of patients with oral cavity cancer in Germany difficult. Nonetheless, the overall lack or very limited progress of 5-year relative survival for patients with oral cavity and pharyngeal cancers emphasize the pivotal importance of enhanced efforts of primary prevention to limit the burden of these cancers, as a very large proportion of them are smoking related and thus, in principle, avoidable.

Further limitations of the present study should be mentioned. First, the small number of observations for lip cancer in early life years did not enable us to provide survival estimates for more narrowly defined age groups. Second, the proportion of cases first notified by death certificates (7.3%) appears relatively high. This seems partly attributable to the unfavorable prognosis of patients with oral cavity cancer and the fact that several of the included cancer registries have been established only recently, implying that some patients were diagnosed with cancer prior to but died after the respective registry was instituted. As DCO cases were excluded from our analysis, survival estimates may have been slightly overestimated [31]. Third, cancer survival could also be under the influence of different treatment approaches used in oral cancer. For example, differential application of elective and therapeutic selective neck dissection could have an impact on survival because neck metastasis is considered an important prognostic factor in oral carcinomas [32]. In this respect, however, the present study is limited because our data base does not include treatment information. Moreover, for a considerable proportion of patients (43.3%) cancer stage was unknown. Such a level of incomplete/missing tumor stage information is not uncommon in population-based registries for oral cavity cancer [33]. In an attempt to explore the extent to which availability of information about tumor stage is relevant, we have calculated age-standardized survival for patients with known and with unknown cancer stage. The aggregate estimates for oral cavity cancer (ICD-10: C00-C06) were 51.4% (SE = 1.2) for patients with known tumor stage and 57.7% (SE = 1.2) for patients with unknown tumor stage, suggesting that the latter subgroup may comprise a relatively large proportion of patients with favorable tumor stages. As we also obtained similar results for separate subsites, our findings should, hence be considered with some caution. Finally, our data base was restricted to eleven out of 16 German Federal States and this may raise some concern of limited validity for the entire German population. Nevertheless, all major regions of Germany (e.g. East – West) were represented approximately according to their population share, and the coverage of about 33 million persons living in Germany enabled a not hitherto available level of precision of survival estimates even for less common subgroups of cancers of the oral cavity.

To summarize, the present study is the first to provide comprehensive information about five-year relative survival of oral cavity cancer patients of almost half of the German population. Patients with cancers of the lip (ICD-10: C00) were shown to have a five-year age-adjusted relative survival of 86.5%. The survival with a cancer of the tongue (ICD-10: C01-02) amounted to 48.1%. Patients with cancer at other regions of the oral cavity (ICD-10: C03-06) had a 5-year relative survival of 51.7%. These survival estimates were similar to those observed for several Scandinavian countries but lower than those recently reported from the US. Our findings further emphasize the high relevance of primary prevention and early detection of oral cavity cancer.
